# Pterygium combined with corneal perforation: a case report

**DOI:** 10.1186/s12886-023-03084-z

**Published:** 2023-08-28

**Authors:** Tianqi Yang, Sun Yuet Chan, Jie Liu, Zhiwei Chen, Guocheng Yu, Xiaojuan He, Jing Meng

**Affiliations:** Department of Ophthalmology, The First Affiliated Hospital of Jinan University, Guangzhou, 510632 China

**Keywords:** Pterygium, Corneal perforation, Autologous serum eye drops, Case report

## Abstract

**Background:**

Pterygium is a common ocular surface disease. Pterygium combined with corneal perforation is rare.

**Case presentation:**

A 28-year-old female patient visited our outpatient clinic due to sudden onset of blurred vision and increased tearing in her left eye. The visual acuity was 1.0 OD and intraocular pressure (IOP) of 19.5 mmHg for the right eye with no significant abnormalities found in the anterior and posterior segments. The visual acuity of her left eye was 0.06, and IOP was 6.2 mmHg. A triangular vascular membranous tissue was seen in her left eye below the nose growing into the cornea and the pupil area was not touched. Slit-lamp examination revealed a tiny round corneal perforation in 8 o’clock position of the lesion area. Hospital diagnosis was given as pterygium combined with corneal perforation. The patient was treated with levofloxacin eye drops and autologous serum-based eye drops.

**Conclusions:**

We report a rare case of pterygium combined with corneal perforation. Perforation is a very rare complication of pterygium. This patient received proper treatment and good result was seen. This article aimed to improve clinicians’ understanding of pterygium.

## Background

Pterygium is a common ocular surface disease symbolized by the corneal invasion of the triangular proliferation of local bulbar conjunctiva and underlying fibrovascular tissue. The most common cause of the disease is ultraviolet radiation, and a shortage of corneal limbal stem cells is one of the main causative factors [1]. Keratoconus [2], Sjogren's syndrome [3], ichthyosis [4], Peters' disease [5], atopic keratoconjunctivitis [6], and Terrien's marginal degeneration [7] are all known for causing spontaneous corneal perforation. Only one similar case has been reported worldwide [8].

## Case presentation

**Fig. 1:**
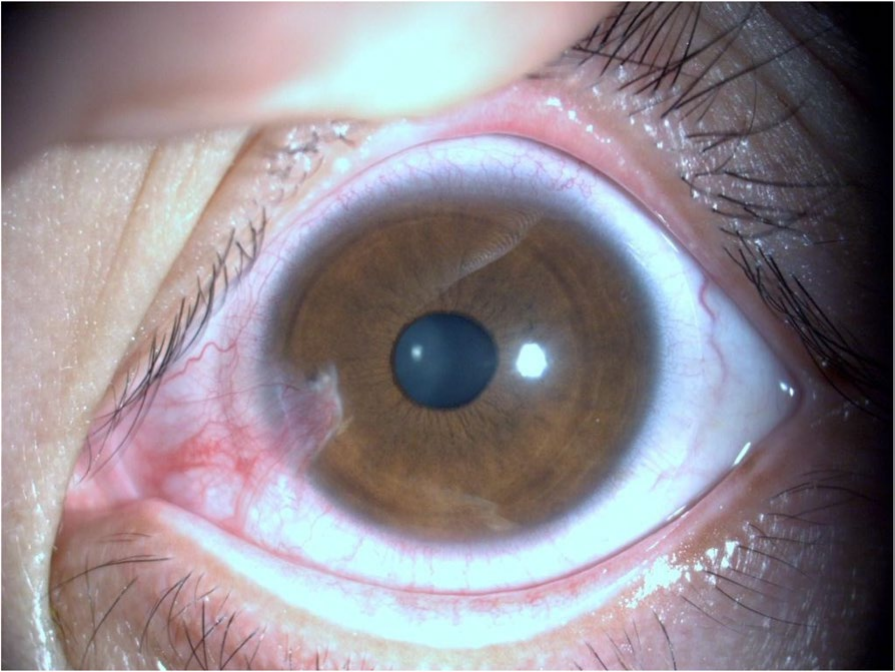
A pterygium tissue was located at 8 o'clock and did not touch the pupillary area

**Fig. 2:**
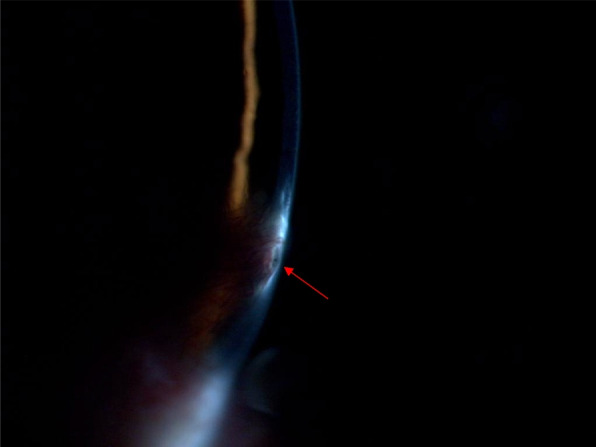
Slit lamp examination revealed a tiny round corneal perforation at the location of the head of the pterygium tissue

**Fig. 3:**
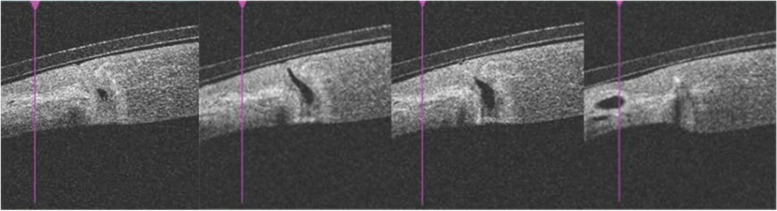
AS-OCT showed that the perforation was located at the junction of the lesion and normal cornea, with high signal in the stroma and thinning of the cornea in the area of the lesion, covered by a corneal bandage lens

The patient was a 28-year-old female. She presented to our ophthalmology department with a 3-day history of sudden onset of blurred vision and increased tearing in her left eye. She had a history of "burn" for 22 years and complained of an injury to her left eye, but the specific treatment was hard to recall. Ophthalmologic examination revealed a visual acuity of 20/20 OD and intraocular pressure (IOP) of 19.5 mmHg, with no significant abnormalities found in the anterior and posterior segments. The visual acuity of her left eye was 20/333, and IOP was 6.2 mmHg. The conjunctiva was slightly congested, a triangular vascular membranous tissue was seen below the nasal site growing into the cornea and the pupil area was not touched (Fig. 1). Slit-lamp examination revealed a tiny round corneal perforation in 8 o’clock position of the lesion area (Fig. 2). The Siedel test was positive, indicating a shallow anterior chamber. Anterior segment optical coherence tomography (AS-OCT) of the left eye showed that the perforation was located at the junction of the lesion and the normal cornea, with a high signal in the stroma and thinning of the cornea in the lesion area (Fig. 3).

Due to the inconvenience of transportation, the patient chose to be hospitalized. Before admission, the patient was treated with levofloxacin eye drops for 2 h. Her left eye was covered by a bandage contact lens. After admission, autologous serum-based eye drop was used frequently (qh) to adjust the ocular surface immunity.

**Fig. 4:**
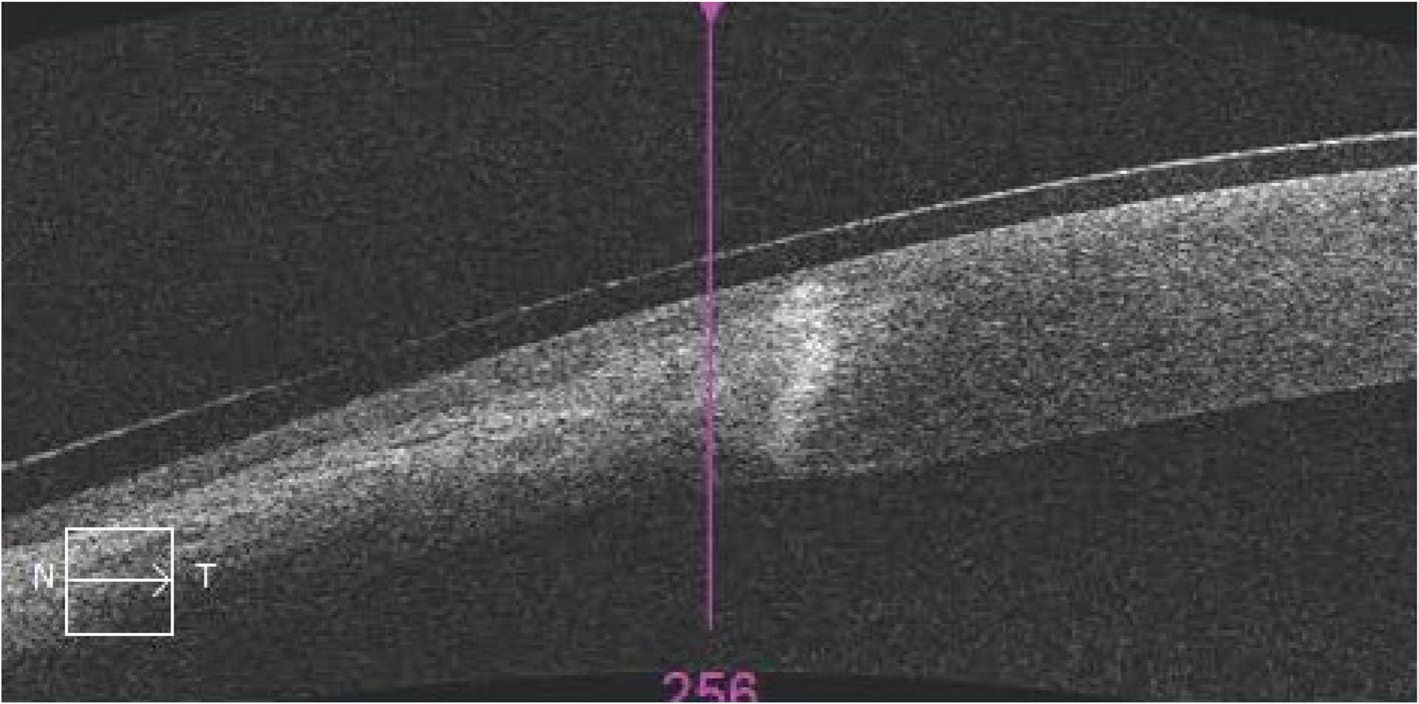
The corneal perforation healed after treatment with autologous serum eye drops, and AS-OCT revealed a residual high-signal scar in the corneal stroma

**Fig. 5:**
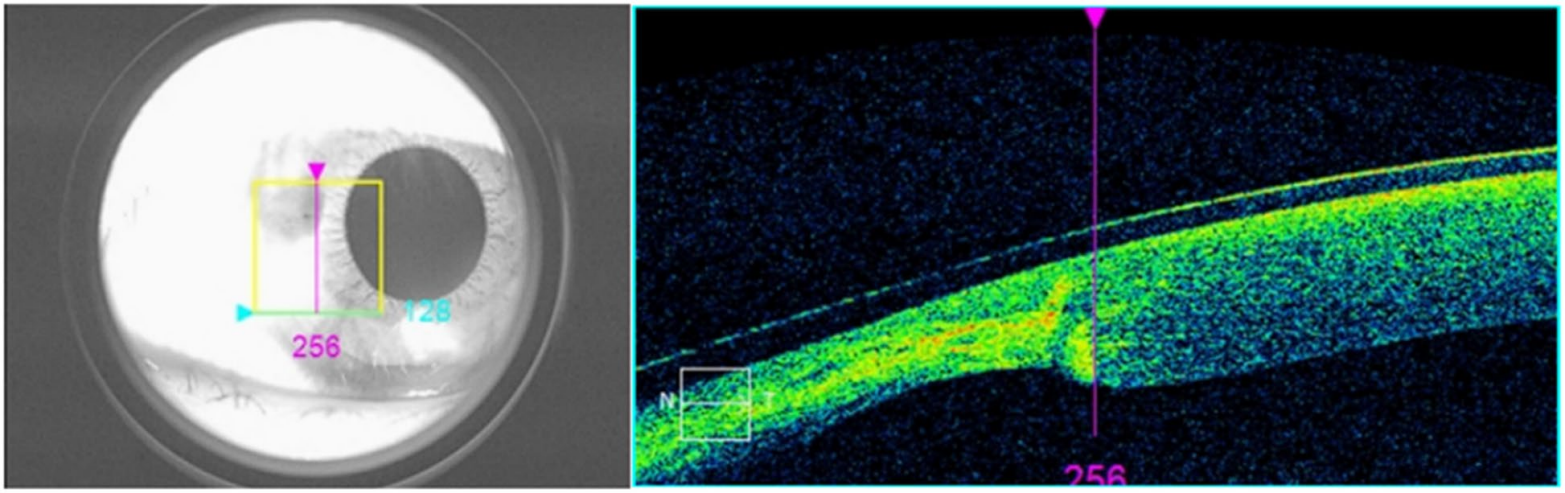
The corneal thickness of the lesion area is thinner than that of the normal cornea

The patient was discharged after a total of 7 days of treatment. At the time of discharge, the left eye had an uncorrected visual acuity of 20/25 and an IOP of 17.0 mmHg. The congestion of the vascular membranous tissue on the nasal side of the cornea had subsided more than before, the corneal perforation was closed, and a high-signal stromal scar was visible on AS-OCT (Fig. 4). The corneal thickness of the lesion area was significantly thinner (Fig. 5).

## Discussion and conclusions

The patient had a clear history of burns, and the eye examination confirmed no apparent iris adhesion or incarceration, indicating that the previous burns did not cause corneal dissolution and perforation directly, but may have damaged the corneal and conjunctival tissues to some extent, injured the limbal stem cells, and damaged the barrier function. The pterygium tissue's blood vessels were large and, the inflammatory reaction was noticeable when the patient was admitted to the hospital. According to studies [9], Matrix metalloproteinases are widely expressed in pterygium, especially in the head. MMPs are a kind of extracellular matrix enzyme that can degrade various extracellular matrix proteins and contribute to tissue remodeling. The corneal stroma, which most types I collagen, accounts for almost 90% of the overall corneal thickness. MMP-2 and MMP-9 are widely expressed in pterygium fibroblasts [10]. MMP-2 can cause the Descemet's membrane to dissolve, whereas MMP-9 can degrade the corneal stroma, thinning the corneal.

Furthermore, tumor necrosis factor-alpha (TNF-α) expression is up-regulated in pterygium tissue and tears of pterygium patients [11]. TNF-α may increase the expression of MMPs in corneal fibroblasts while diminishing the expression of tissue inhibitors of metalloproteinases [12], resulting in an imbalance between the two expressions that leads to corneal stroma degradation and further reduces the mechanical stability of the cornea. Mechanical stimulation has been proven to enhance the expression of inflammatory factors and proteases, speed up matrix degradation [13], and cause a vicious cycle. The patient's AS-OCT showed significant corneal thinning in the lesion area, which was consistent with the pterygium head's position, and the capacity to resist intraocular pressure in this area was decreased. After taking autologous serum to modulate immunity, the inflammatory reaction reduced and the corneal perforation recovered, indicating that local inflammation was a crucial factor in the progression of the patient's disease. Therefore, previous burns resulted in the lack of local limbal stem cells, secondary pterygium-induced corneal stroma remodeling, and further reduced corneal stability under the action of inflammatory factors, resulting in a reduced ability to resist intraocular pressure, which may be a reasonable explanation for spontaneous corneal perforation in this patient.

Corneal perforation is a common acute severe ophthalmology condition that can substantially impair the injured eye's visual function. Surgical treatment is the most common treatment, yet individualized treatment can benefit patients more depending on the location, etiology, size, and other factors. The autologous serum-based eye drop (ASED) is a light-yellow transparent liquid prepared by centrifuging autologous venous blood after in vitro coagulation. It's packed with growth factors, vitamins, and other essential elements. It can assist corneal epithelial cells to grow and proliferate while keeping the ocular surface microenvironment stable [14]. ASED have been shown to modulate stromal corneal wound-healing activities by controlling matrix metalloproteinase activity [15]. In addition, Fibronectin present in serum plays an important role in the process of ocular surface reepithelization. In animal models, topical application of fibronectin promotes epithelial wound healing by promoting epithelial migration [16]. Autologous serum eye drops used in eye spot is a very effective method for the treatment of corneal and ocular surface diseases. It can provide a large number of active substances (epidermal growth factor (EGF), vitamin A, transforming growth factor B(TGF-β), etc.), simulate the function of natural tears, promote the proliferation, migration and differentiation of corneal and conjunctival epithelial cells, and have been shown to be safe, effective and well tolerated in numerous studies [17]. At present, there is no uniform standard for the preparation and use of autologous serum eye drops in the world. Commonly used concentrations are 20%, 50% and 100%, the treatment frequency ranges from 4x /day to hourly usage [18]. There was a published study [19] that showed 100% ASED is the most effective in decreasing symptoms, corneal epitheliopathy and promoting fast closure of wound.

Given the thinning of the corneal thickness in the corneal perforation area in this patient, pterygium excision is dangerous and may result in iatrogenic injury. After comprehensive consideration of the risks and benefits as well as the patient's personal wishes, we chose conservative treatment, which used high concentration (100% serum) autologous serum frequently (qh) in the initial stage to regulate the immune system, combined with bandage mirror covering and pressure bandaging, and achieved good results.

## Data Availability

The datasets used and/or analyzed in the course of the current study are available from the corresponding author on reasonable request.

## Abbreviations

AS-OCT: Anterior segment optical coherence tomography
IOP: Intraocular pressure
MMPs: Matrix metalloproteinases
TNF-α: Tumor necrosis factor-alpha
ASED: Autologous serum-based eye drop
